# Supplementary material for: Ubiquitin pathway blockade reveals endogenous ADP-ribosylation marking PARP7 and AHR for degradation
Source: EMBO J. 2025 Dec 1;45(1):261–77. doi: 10.1038/s44318-025-00656-1 (PMC12759070; doi:10.1038/s44318-025-00656-1)
Supplement: Supplementary file 1 — Appendix [file 44318_2025_656_MOESM1_ESM.pdf]

Appendix for:

Ubiquitin pathway blockade reveals endogenous ADP-ribosylation marking PARP7 and AHR for degradation

Andrii Gorelik[1*], Nina Đukić[1], Rebecca Smith[1], Chatrin Chatrin[1], Osamu Suyari[1], Jason Matthews[2,3] and Ivan Ahel[1*]

1 Sir William Dunn School of Pathology, University of Oxford, Oxford, OX1 3RE, UK

2 Department of Nutrition, Institute of Basic Medical Sciences, University of Oslo, 0317 Oslo, Norway

3 Department of Pharmacology and Toxicology, University of Toronto, Toronto, ON M5S 1A8, Canada

*Correspondence: andrii.gorelik@path.ox.ac.uk and ivan.ahel@path.ox.ac.uk

Table of contents

Appendix Figure S1. Titration of TAK243. 2

Appendix Figure S2. Inhibition of the endoplasmic reticulum (ER) stress does not affect the TAK243/MG132-induced ADPr. 3

Appendix Figure S3. ADPr signal induced by TAK243 corresponds to PARP7 and AHR. 4

Appendix Figure S4. AHR band shift induced by the tapinarof and TAK243 co-treatment is ADPr-dependent. 5

Appendix Figure S5. Validation of the siRNA knockdown efficiency. 6

Appendix Figure S6. Validation of the ADPr signal specificity upon siRNA knockdown of DTX2 and RNF146. 7

Appendix Figure S7. Validation of ubiquitin linkages induced by DTX2 and MG132/TAK243 on AHR and other ADPr substrates. 8

Appendix Figure S8. DTX2 interacts with the ADP-ribosylated AHR. 9

Appendix Figure S9. Ubiquitin levels change in the nucleus upon TAK243/tapinarof treatments. 10

Appendix Table S1. qPCR primers used in the study. 12

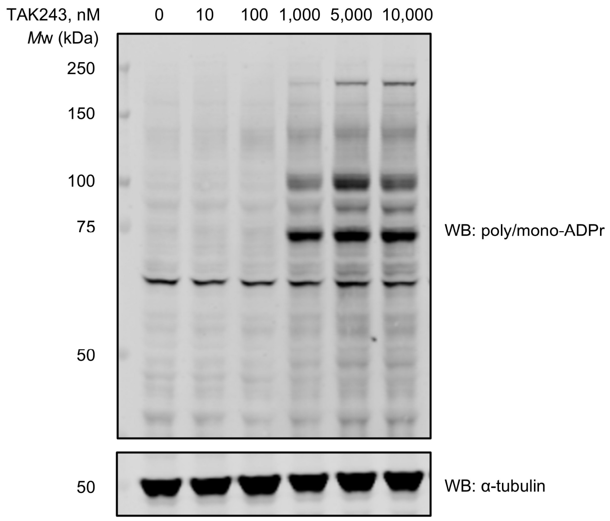

Appendix Figure S1. Titration of TAK243. HCC44 cells were treated with DMSO or TAK243 at varying concentrations for 4 h. ADPr signal was assessed by western blotting using the anti-poly/mono-ADPr antibody, with α-tubulin as a loading control.

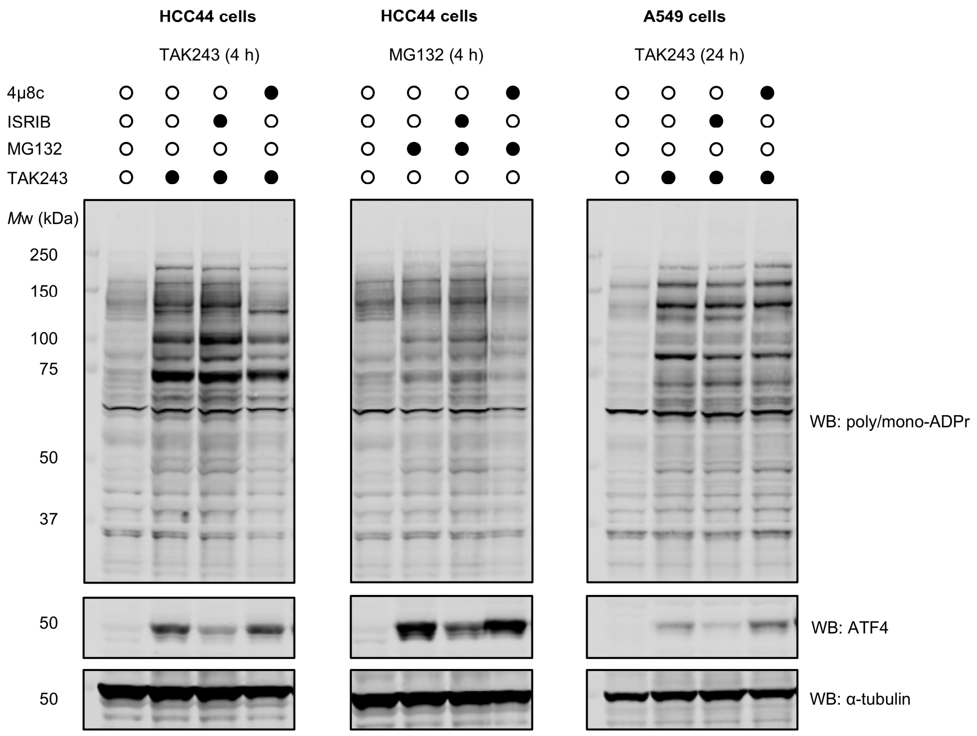

Appendix Figure S2. Inhibition of the endoplasmic reticulum (ER) stress does not affect the TAK243/MG132-induced ADPr. HCC44 cells were pre-treated with DMSO, ISRIB (10 µM) or 4µ8C (10 µM) for 20 h and further treated with MG132 (10 µM) or TAK243 (1 µM) for 4 h. A549 cells were treated with DMSO, TAK243 (1 µM), ISRIB (10 µM) or 4µ8C (10 µM) for 24 h. ADPr signal was assessed by western blotting using the anti-poly/mono-ADPr antibody. ATF4 was used as an ER stress marker with α-tubulin as a loading control.

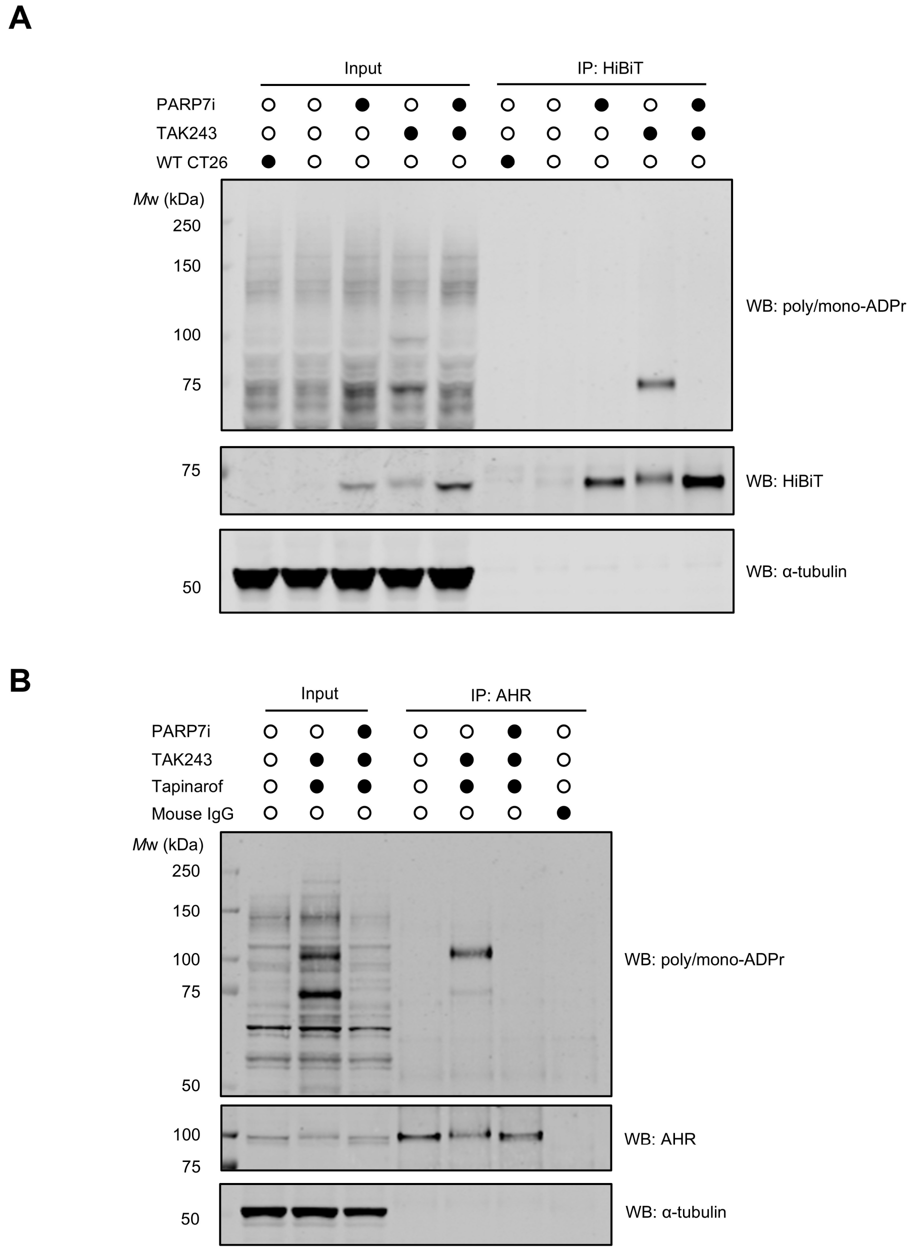

Appendix Figure S3. ADPr signal induced by TAK243 corresponds to PARP7 and AHR. **(A)** PARP7 was immunoprecipitated from HiBiT-PARP7 CT26 cell lysates (treated with DMSO, 1 μM TAK243 and PARP7i for 4 h). Wild type CT26 cells were used as a negative control. PARP7 was detected with an anti-HiBiT antibody. **(B)** AHR was immunoprecipitated from HCC44 cell lysates (treated with DMSO, 1 μM TAK243, 100 nM PARP7i (RBN2397) and 1 μM tapinarof for 4 h. Mouse IgG was used as a negative control for the pulldown. AHR was detected with an anti-AHR antibody. ADP-ribosylation was assessed by western blotting using the anti-poly/mono-ADPr antibody. α-tubulin was used as a loading control.

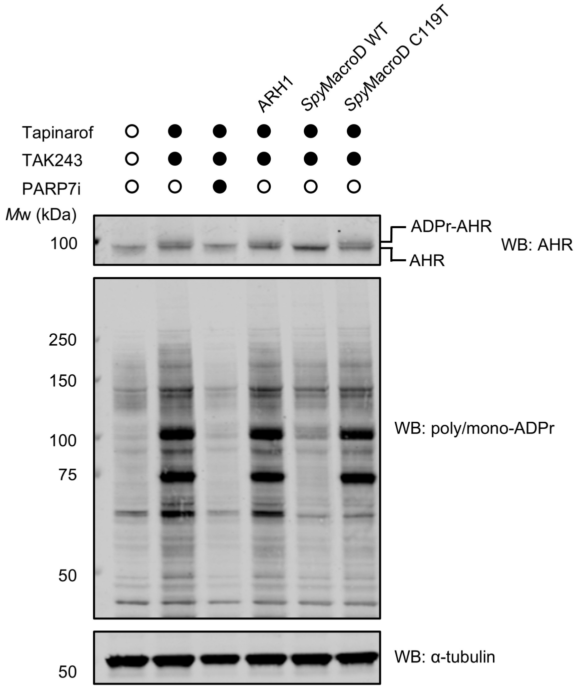

Appendix Figure S4. AHR band shift induced by the tapinarof and TAK243 co-treatment is ADPr-dependent. *Streptococcus pyogenes* MacroD hydrolase treatment was performed on HCC44 cell lysates for 2 h at 37 ˚C. *Spy*MacroD C119T is an inactive mutant of *Spy*MacroD. ARH1 hydrolase treatment was used as a control. ADP-ribosylation was assessed by western blotting using the anti-poly/mono-ADPr antibody. AHR was detected with an anti-AHR antibody with α-tubulin as a loading control.

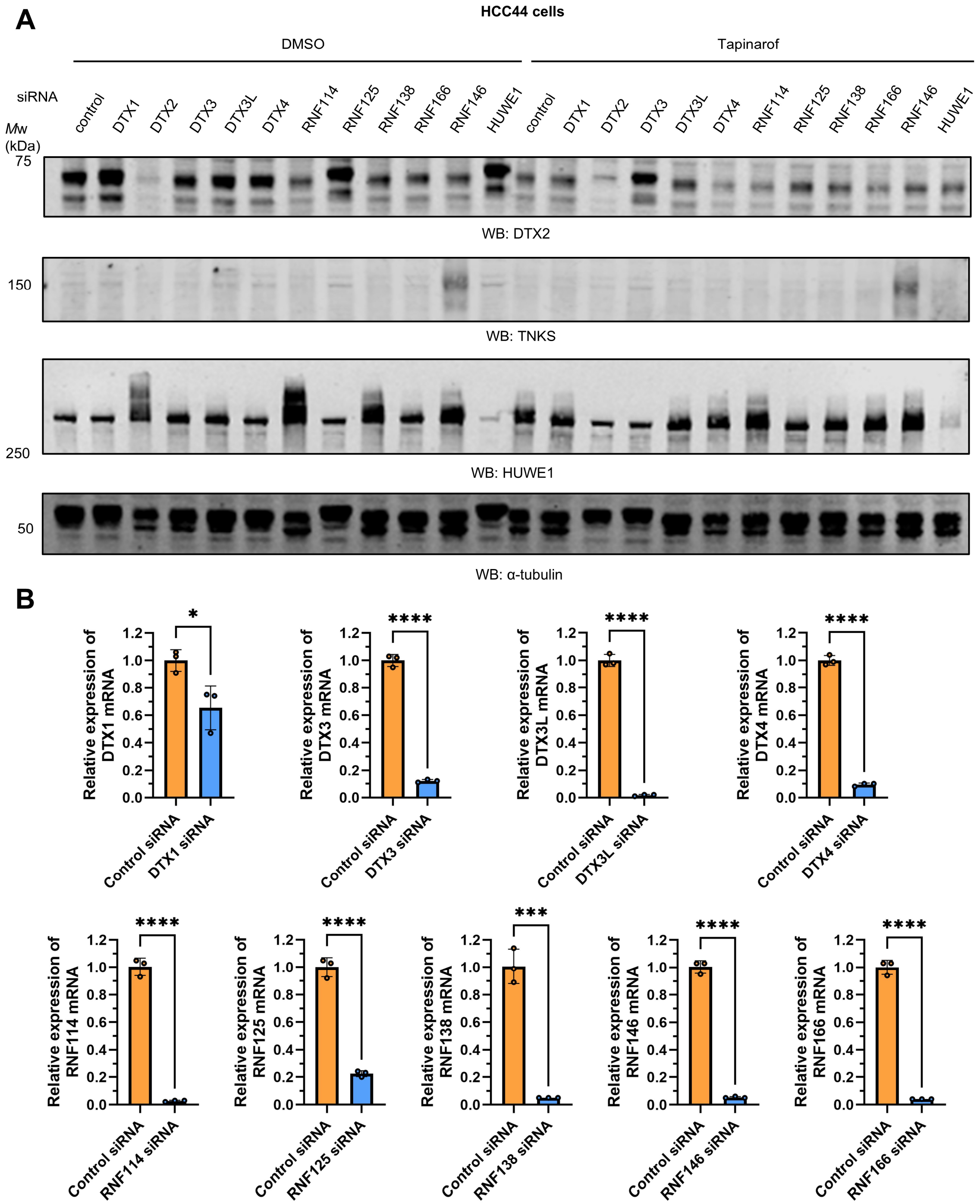

Appendix Figure S5. Validation of the siRNA knockdown efficiency. (A) HCC44 cells were transfected with a panel of siRNAs. DTX2 and HUWE1 knockdown efficiency was assessed by western blotting using the corresponding antibodies. RNF146 knockdown was assessed by blotting for TNKS1/2. α-tubulin was used as a loading control. **(B)** Knockdown of DTX1, DTX3, DTX3L, DTX4, RNF114, RN125, RN138, RNF146, RNF166 was assessed by RT-qPCR. **P* = 0.0278, ****P* = 0.0002, *****P* < 0.0001, calculated by Student's t-test (two-tailed, unpaired). Data are shown as mean ± s.d. of n = 3 technical replicates.

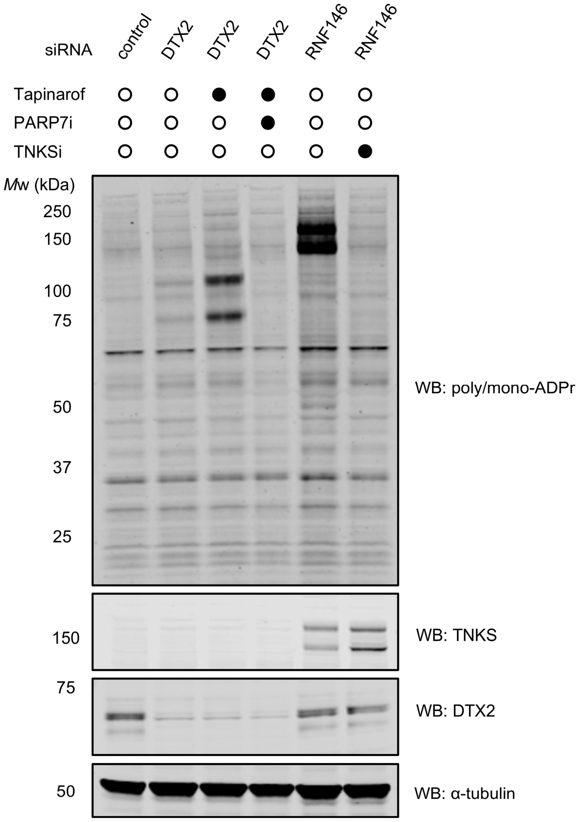

Appendix Figure S6. Validation of the ADPr signal specificity upon siRNA knockdown of DTX2 and RNF146. HCC44 cells were transfected with siRNAs against DTX2 and RNF146 and treated. DTX2 knockdown cells were additionally treated with 1 μM tapinarof alone or in combination with 1 μM PARP7i for 24 h. RNF146 knockdown cells were additionally treated with 1 μM TNKSi for 24 h. DTX2 knockdown efficiency was assessed by western blotting using the corresponding antibody. RNF146 knockdown was assessed by blotting for TNKS1/2. ADPr signal was assessed by western blotting using the anti-poly/mono-ADPr antibody. α-tubulin was used as a loading control.

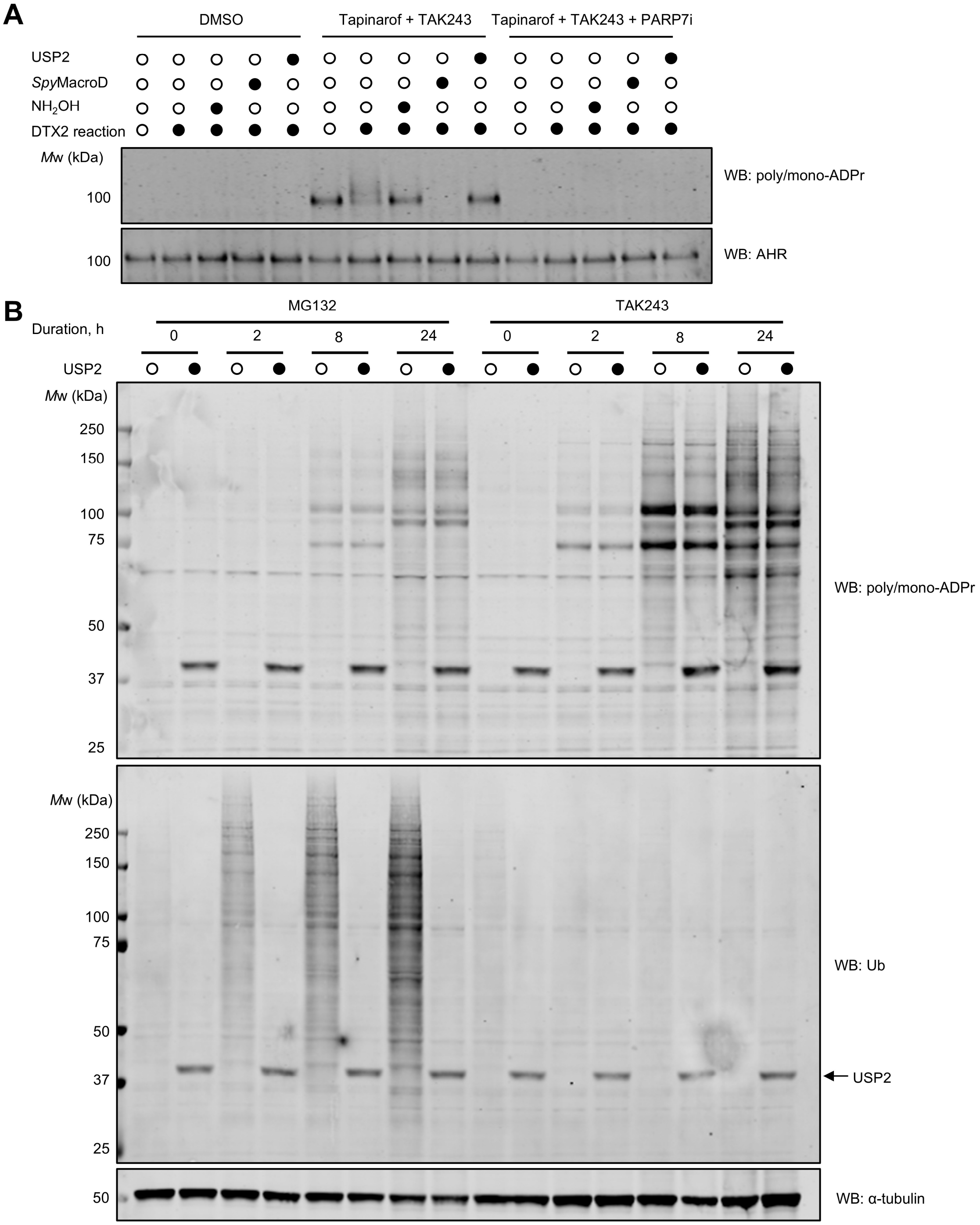

Appendix Figure S7. Validation of ubiquitin linkages induced by DTX2 and MG132/TAK243 on AHR and other ADPr substrates. (A) Immunoprecipitated AHR from HCC44 cell lysates (treated with 1 µM TAK243, PARP7i and tapinarof for 4 h) was incubated with E1/E2 and DTX2. *Spy*MacroD ADPr hydrolase, USP2 deubiquitinase and NH_2OH treatments were performed on the bead-bound AHR**.** AHR was detected with an anti-AHR antibody. **(B)** Cell lysates from Fig. 1C were treated with the USP2 deubiquitinase for 2 h at 37 ˚C. ADP-ribosylation was assessed by western blotting using the anti-poly/mono-ADPr antibody, ubiquitin signal was assessed using an anti-ubiquitin antibody. α-tubulin was used as a loading control.

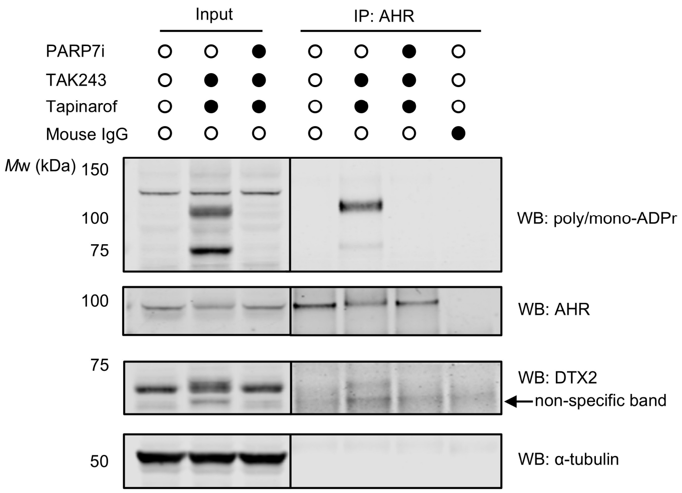

Appendix Figure S8. DTX2 interacts with the ADP-ribosylated AHR. AHR was immunoprecipitated from HCC44 cell lysates (treated with DMSO, 1 µM TAK243, 1 µM PARP7i (RBN2397), and 1 µM tapinarof for 4 h). Mouse IgG was used as a negative control for the pulldown. DTX2 and AHR were detected with an anti-DTX2 and anti-AHR antibodies respectively. ADP-ribosylation was assessed by western blotting using the anti-poly/mono-ADPr antibody. α-tubulin was used as a loading control.

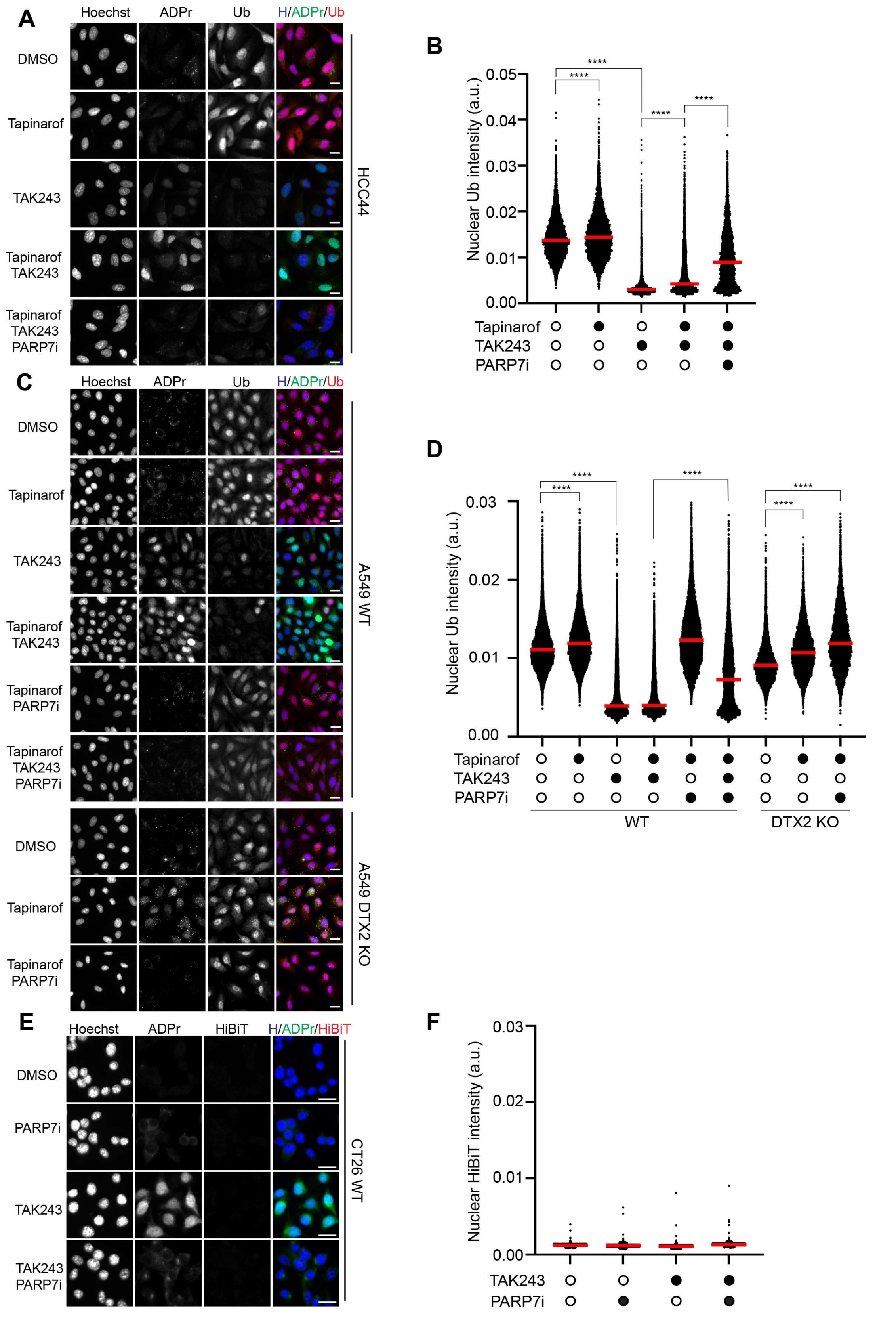

Appendix Figure S9. Ubiquitin levels change in the nucleus upon TAK243/tapinarof treatments. (A) Widefield images showing HCC44 cells treated with 1 µM tapinarof (24 h), 1

μM TAK243 (4 h) and 100 nM PARP7i (RBN2397, 24 h) alone and in combination. Cells were stained with Hoechst (blue), ADPr (poly/mono-ADP ribose, green) and Ub (ubiquitin, magenta). **(B)** Quantification of nuclear Ub from (A). **(C)** Widefield images showing A549 WT or DTX2 KO cells treated with 1 μM tapinarof (24 h), 1 μM TAK243 (4 h) and 100 nM PARP7i (RBN2397, 24 h) alone and in combination. Cells were stained with Hoechst (blue), ADPr (poly/mono-ADP ribose, green) and Ub (Ubiquitin, magenta). **(D)** Quantification of nuclear Ub (C). **(E)** Widefield images showing wild type CT26 cells treated with 1 μM TAK243 (4 h) and 100 nM PARP7i (RBN2397, 24 h) alone and in combination. Cells were stained with Hoechst (blue), ADPr (poly/mono-ADP ribose) (green) and HiBiT (magenta). **(F)** Quantification of nuclear HiBiT (E). For all images, scale bar = 20 μm. Source data are available online for this figure. Statistical analysis was performed using an ordinary one-way ANOVA. Asterisks indicate statistical significance (*****P* < 0.0001). Red bars indicate median for each condition. Source data are available online for this figure.

Gene	Forward primer	Reverse primer
CYP1A1	GATTGAGCACTGTCAGGAGAAGC	ATGAGGCTCCAGGAGATAGCAG
CYP1B1	GAGAACGTACCGGCCACTAT	AAGGAAGGCCAGGACATAGG
HPRT1	GCGTCGTGATTAGCGATGATG	CTCGAGCAAGTCTTTCAGTCC
DTX1	AGAATCCCGAGGATGTGGTTCG	TCGTAGCCTGATGCTGTGACCA
DTX3	GGAGACCGAATGTCATCACCTG	CTGTGATACCCTTCGCTCTCAG
DTX3L	CCAGGTTATGAGTCCTTTGGCAC	TGCAGTTCGCTGTATTCCAGGG
DTX4	GCCACCTTGAATCGTACCAACC	GGTTGACAGGACTGGACCCATT
RNF114	CCGTGTGCTTAGAGGTGTACGA	CAGACAGGCTTCTTCGGCTTCA
RNF125	TGAGGTGTTACACCAGCCTGTC	GGCTACATCAGTTGCTGGAACTC
RNF138	TGTCAGGAGGTGCTCAAAACGC	CTCTTCTAGTCACATTTCCACGAC
RNF146	AGTCAGTCTGCCCTGTAAGCAC	GGCTTGTCAAGGAAATCCTCGG
RNF166	GGTGAAGCACTGTGTGGAAAGC	CGTAGGAGAACTTGTGTCGGTG

Appendix Table S1. qPCR primers used in the study.
