# Supplementary figures and images for: Ubiquitin pathway blockade reveals endogenous ADP-ribosylation marking PARP7 and AHR for degradation
Source: EMBO J. 2025 Dec 1;45(1):261–77. doi: 10.1038/s44318-025-00656-1 (PMC12759070; doi:10.1038/s44318-025-00656-1)

Fig. 1B

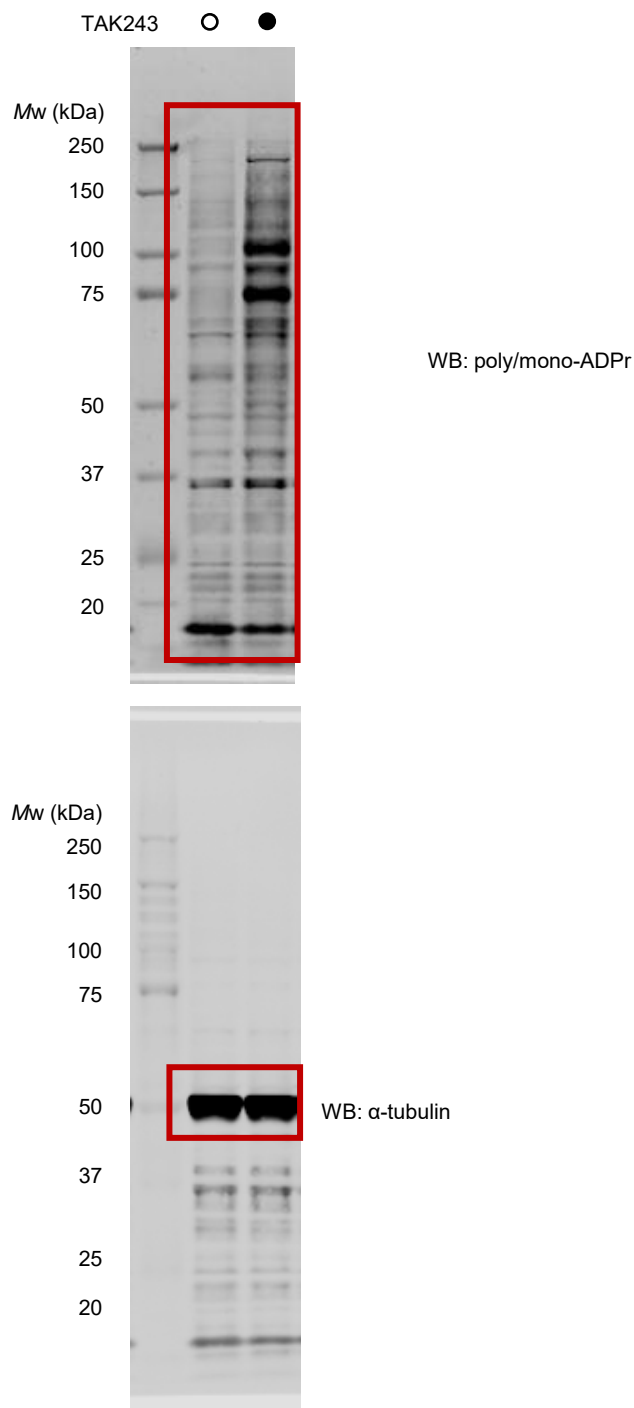

Supplement: Supplementary file 3 — Source data Fig. 1 [file 44318_2025_656_MOESM3_ESM.zip › Figure 1/1B/Fig. 1B.pdf]

Fig.1C

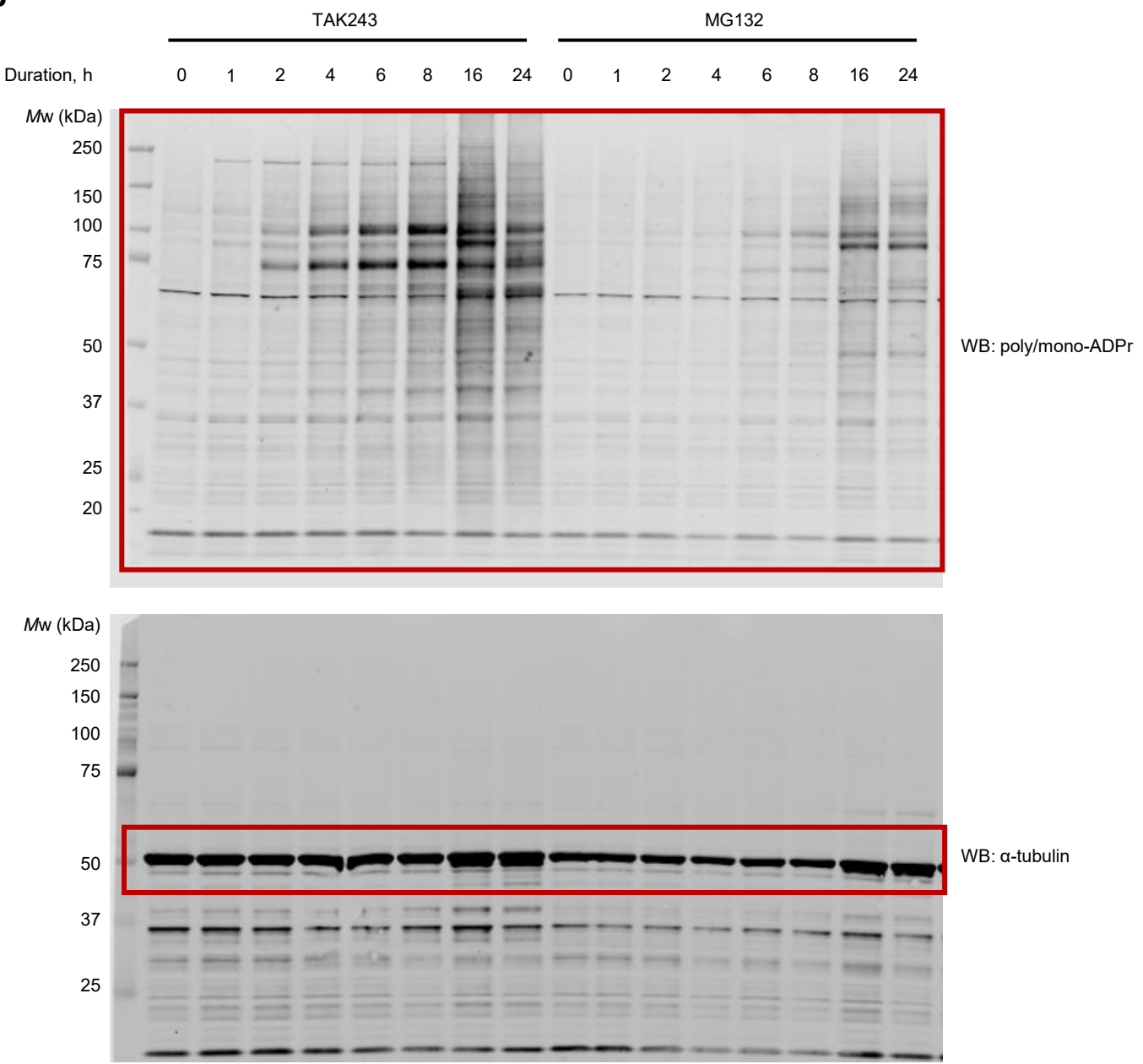

Supplement: Supplementary file 3 — Source data Fig. 1 [file 44318_2025_656_MOESM3_ESM.zip › Figure 1/1C/Fig. 1C.pdf]

Fig.2A

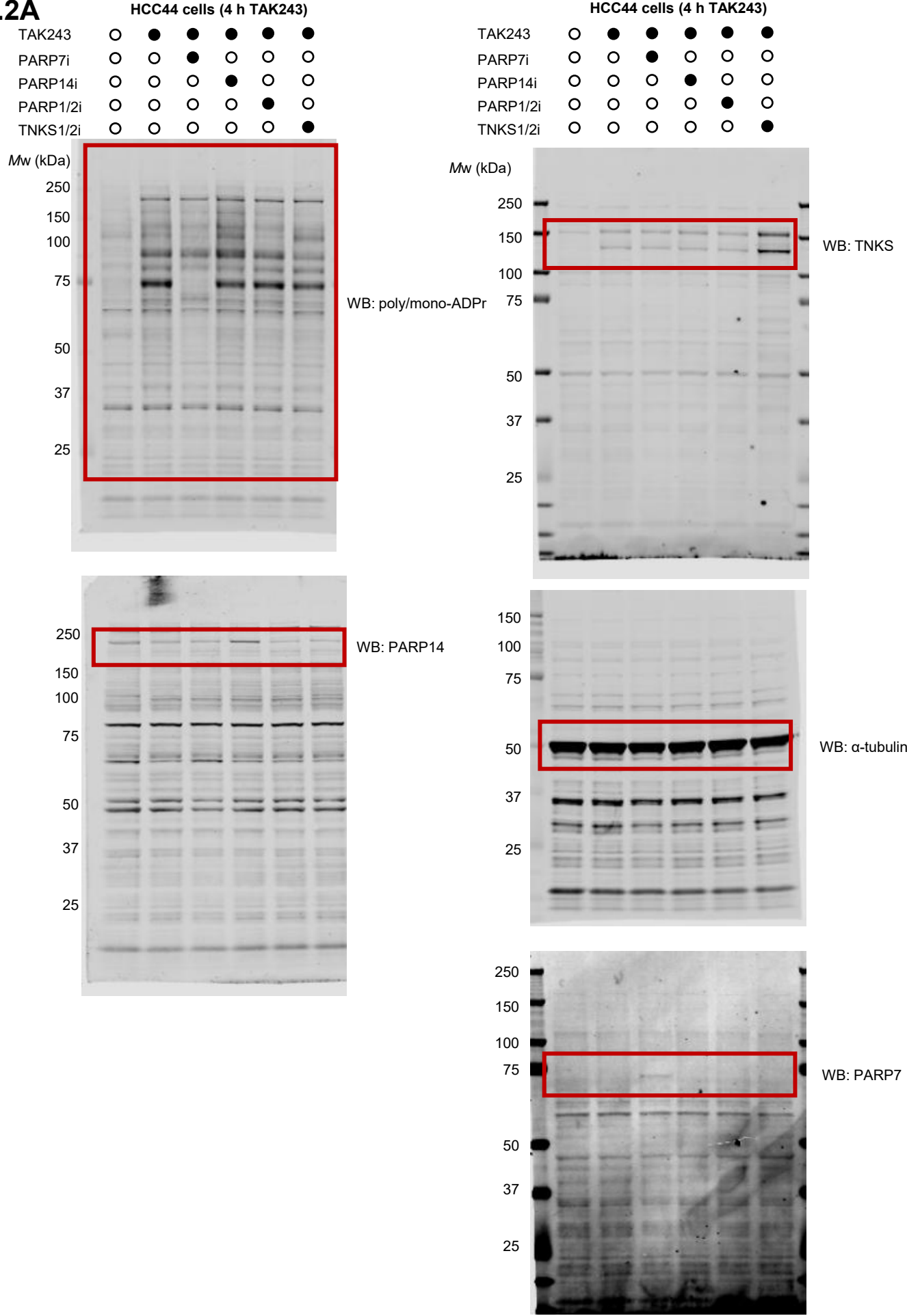

Supplement: Supplementary file 4 — Source data Fig. 2 [file 44318_2025_656_MOESM4_ESM.zip › Figure 2/2A/Fig. 2A.pdf]

Fig. 2B

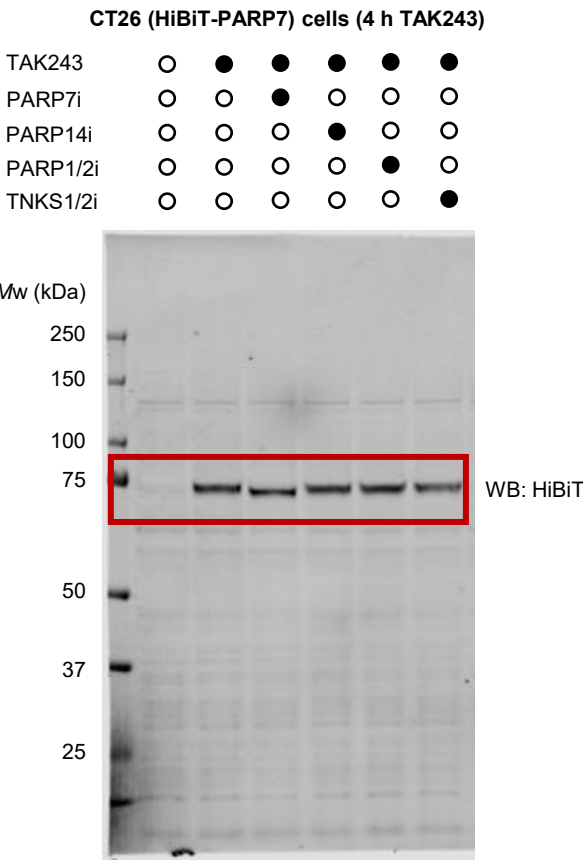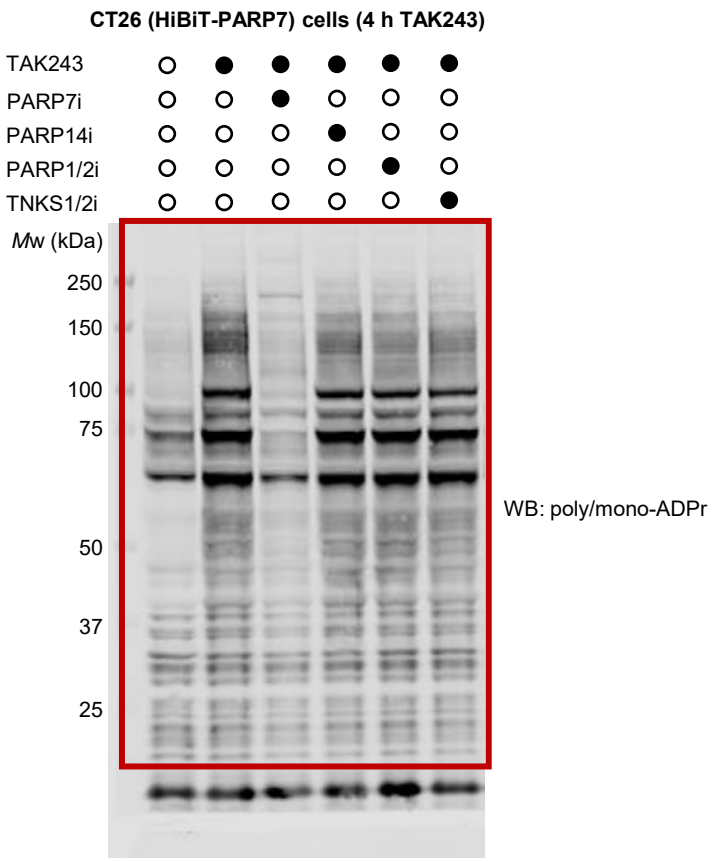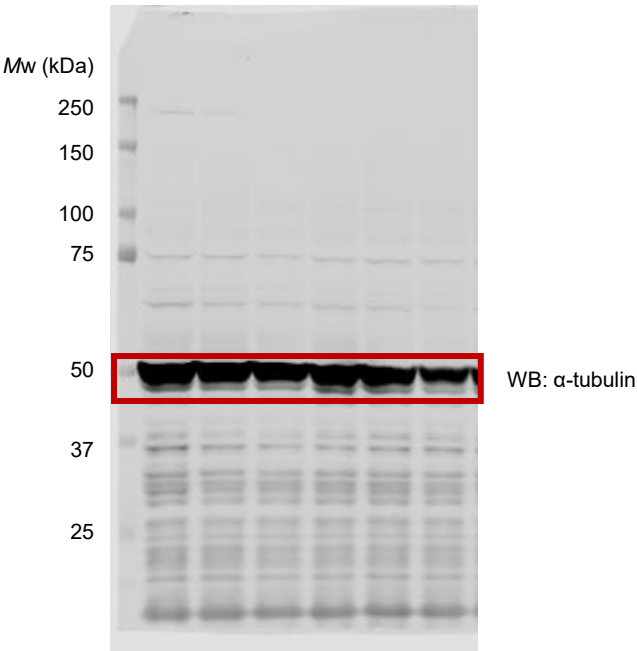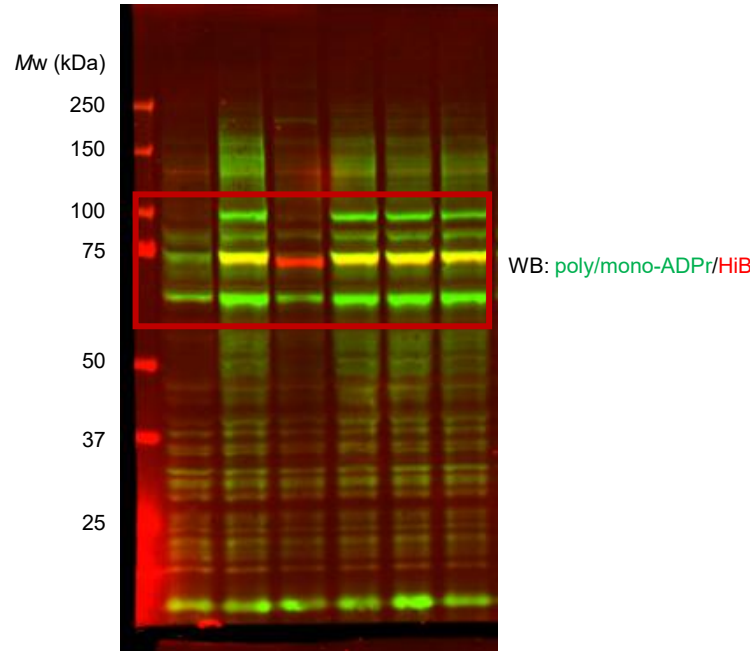

Supplement: Supplementary file 4 — Source data Fig. 2 [file 44318_2025_656_MOESM4_ESM.zip › Figure 2/2B/Fig. 2B.pdf]

Fig. 2D

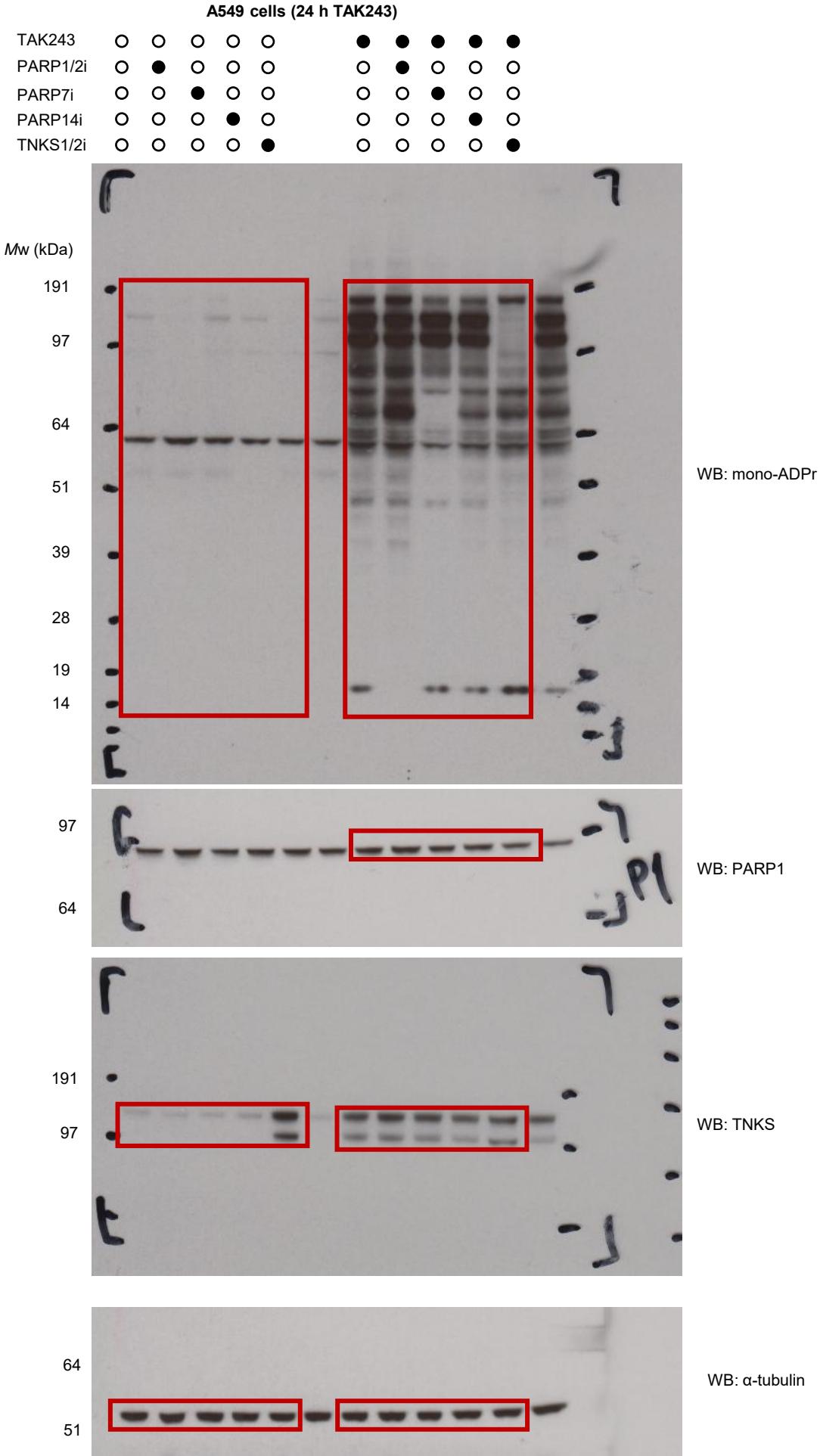

Supplement: Supplementary file 4 — Source data Fig. 2 [file 44318_2025_656_MOESM4_ESM.zip › Figure 2/2D/Fig. 2D.pdf]

Fig. 3C

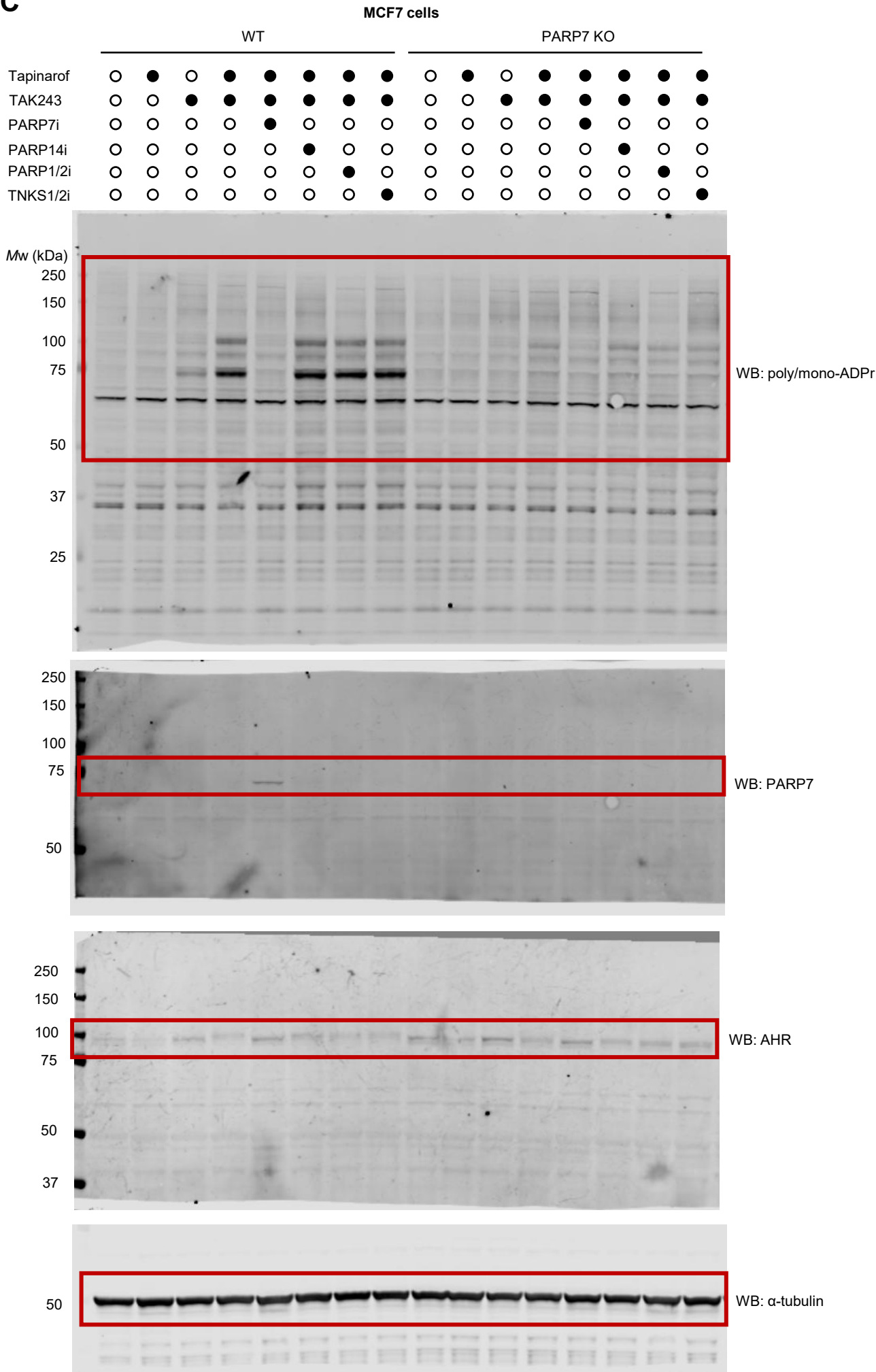

Supplement: Supplementary file 5 — Source data Fig. 3 [file 44318_2025_656_MOESM5_ESM.zip › Figure 3/3C/Fig. 3C.pdf]

**Fig. 4A**

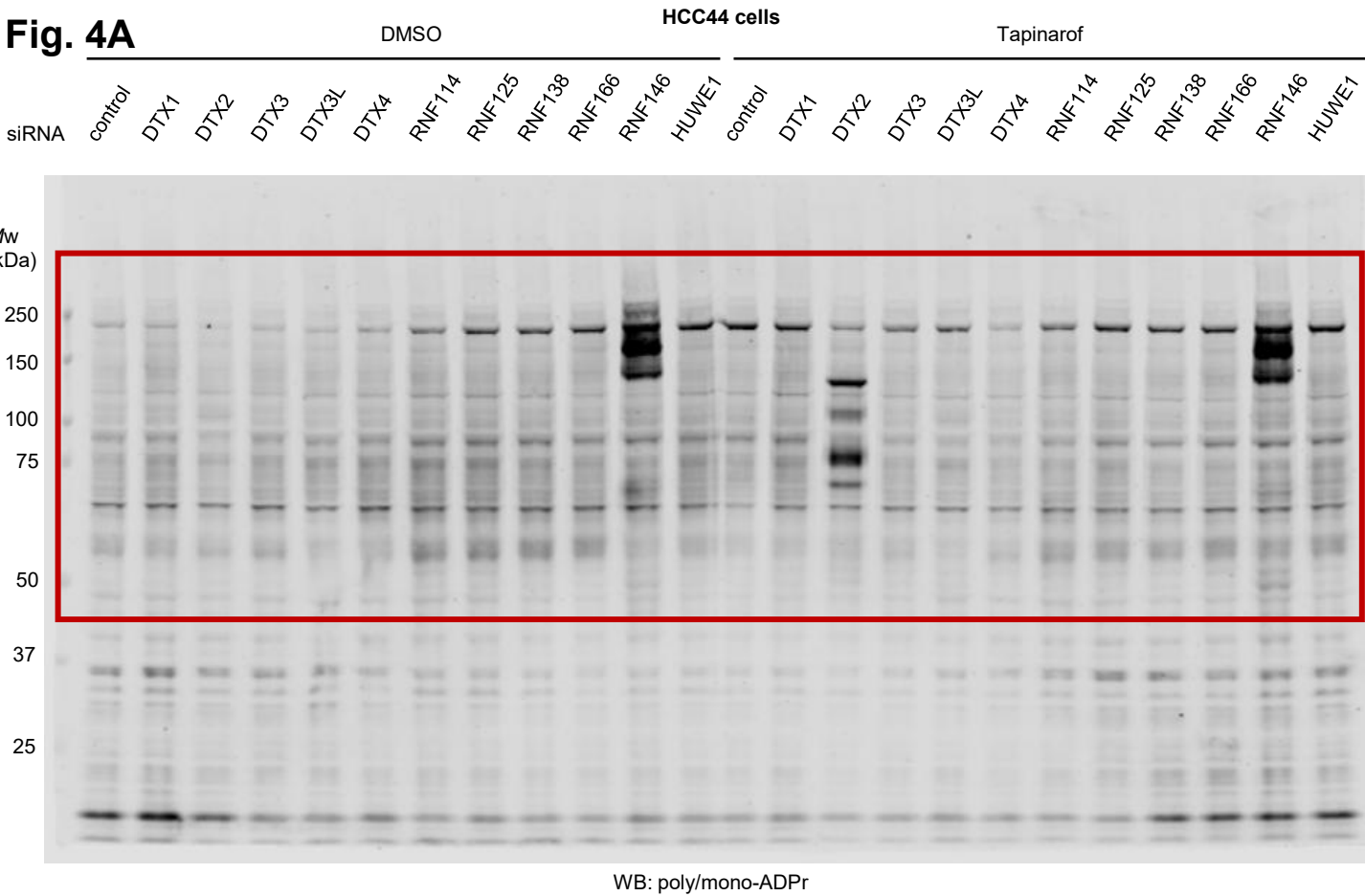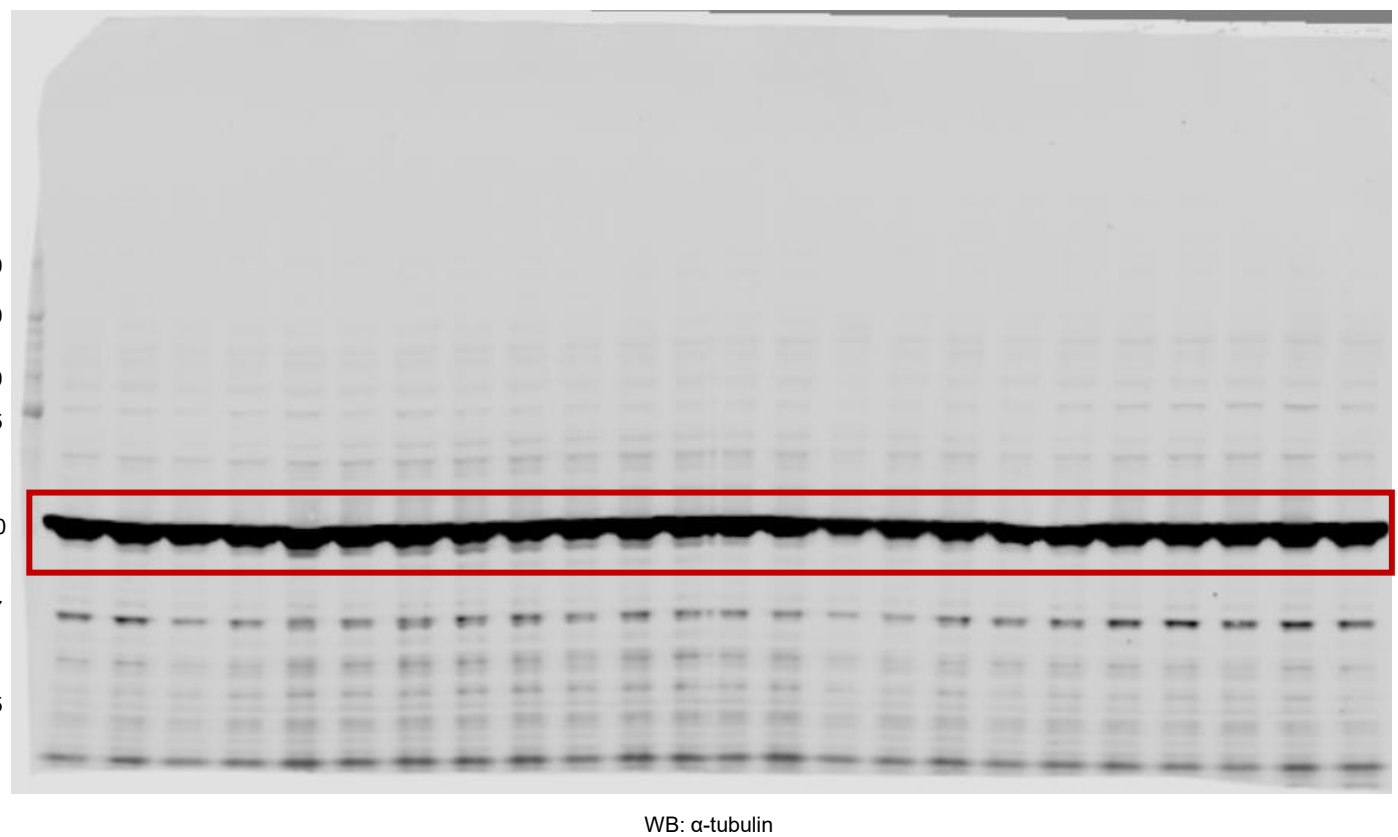

Supplement: Supplementary file 6 — Source data Fig. 4 [file 44318_2025_656_MOESM6_ESM.zip › Figure 4/4A/Fig. 4A.pdf]

Fig. 4B

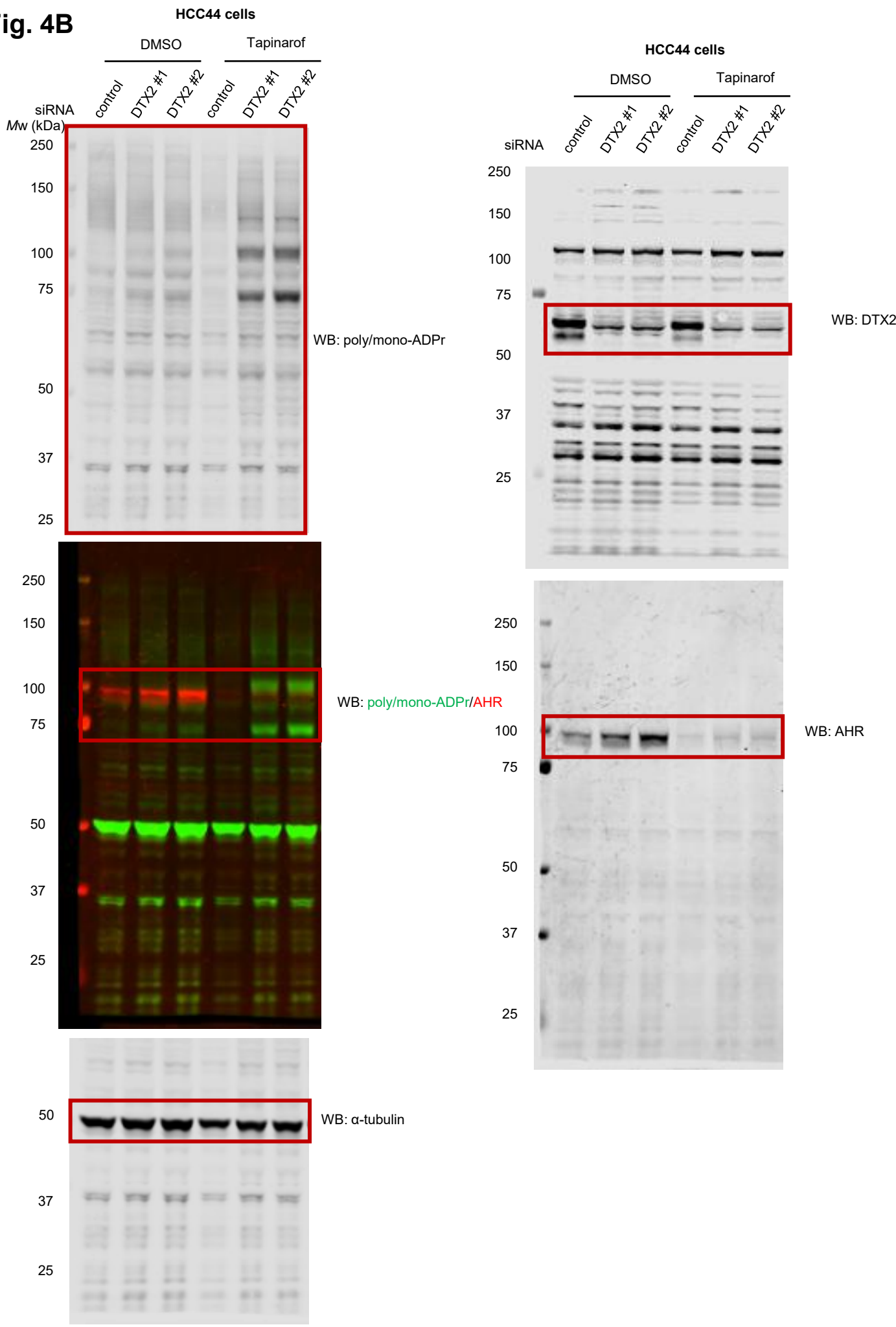

Supplement: Supplementary file 6 — Source data Fig. 4 [file 44318_2025_656_MOESM6_ESM.zip › Figure 4/4B/Fig. 4B.pdf]

**Fig. 4C**

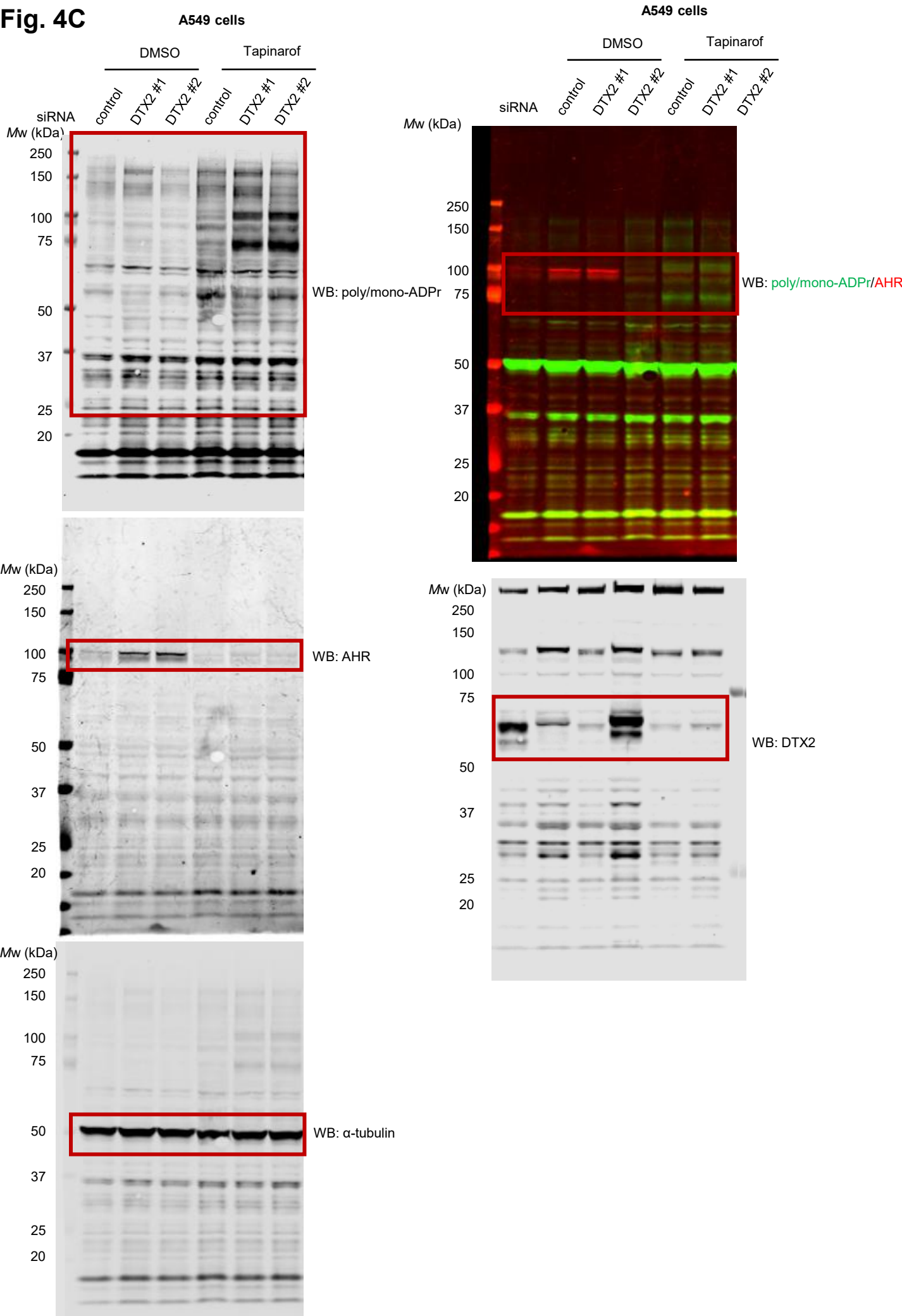

Supplement: Supplementary file 6 — Source data Fig. 4 [file 44318_2025_656_MOESM6_ESM.zip › Figure 4/4C/Fig. 4C.pdf]

**Fig. 5A**

|                  | WT |   |   |   | DTX2 KO |   |   |   |   |
|------------------|----|---|---|---|---------|---|---|---|---|
| Tapinarof (24 h) | ○  | ● | ● | ○ | ○       | ○ | ● | ● | ○ |
| TAK243           | ○  | ○ | ○ | ● | ●       | ○ | ○ | ○ | ● |
| PARP7i           | ○  | ○ | ● | ○ | ●       | ○ | ○ | ● | ○ |

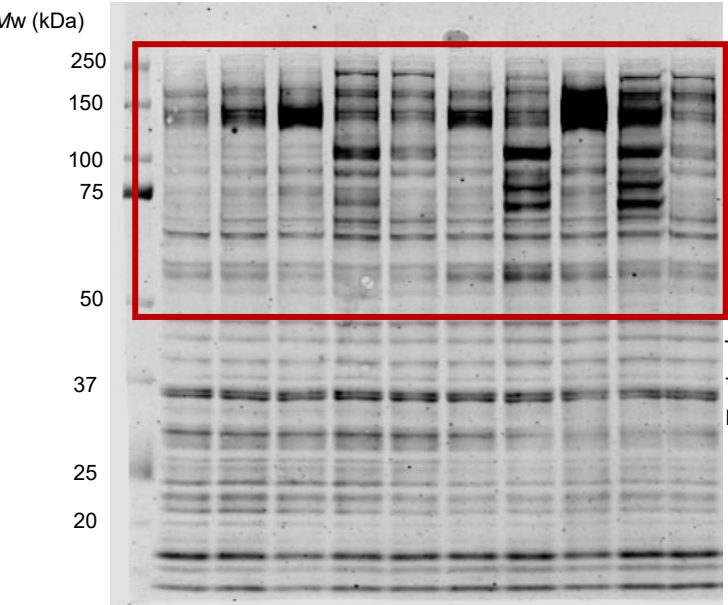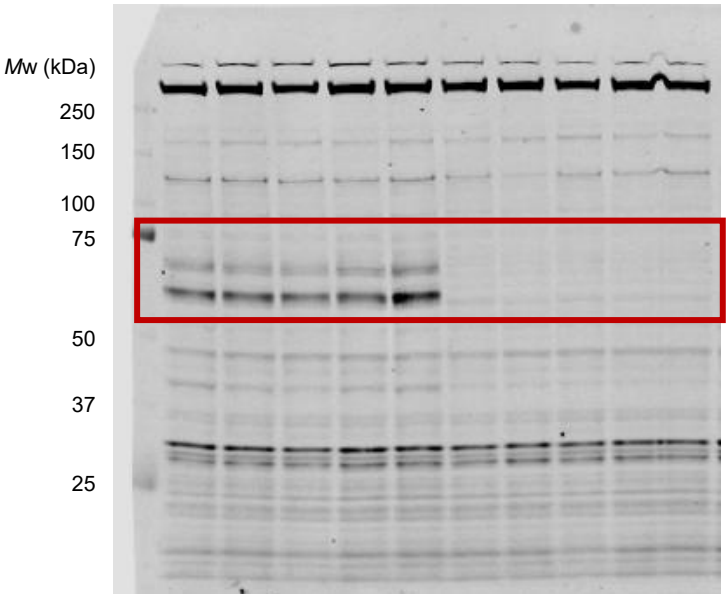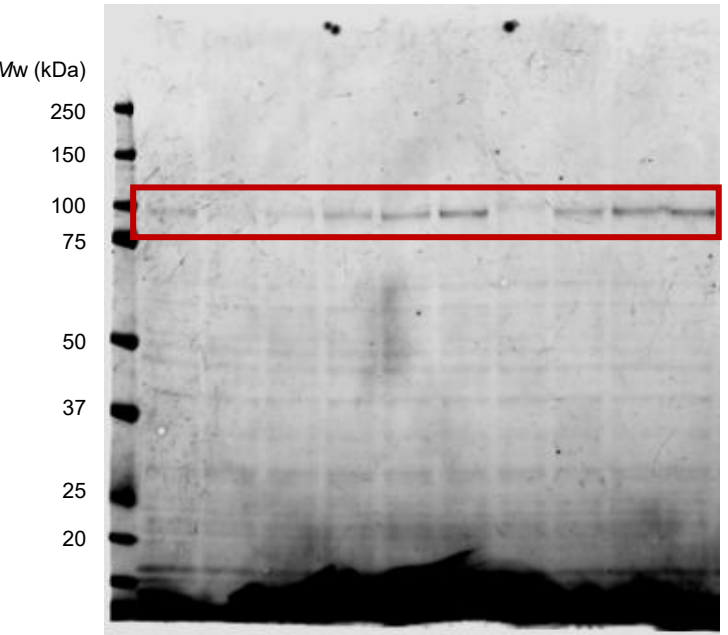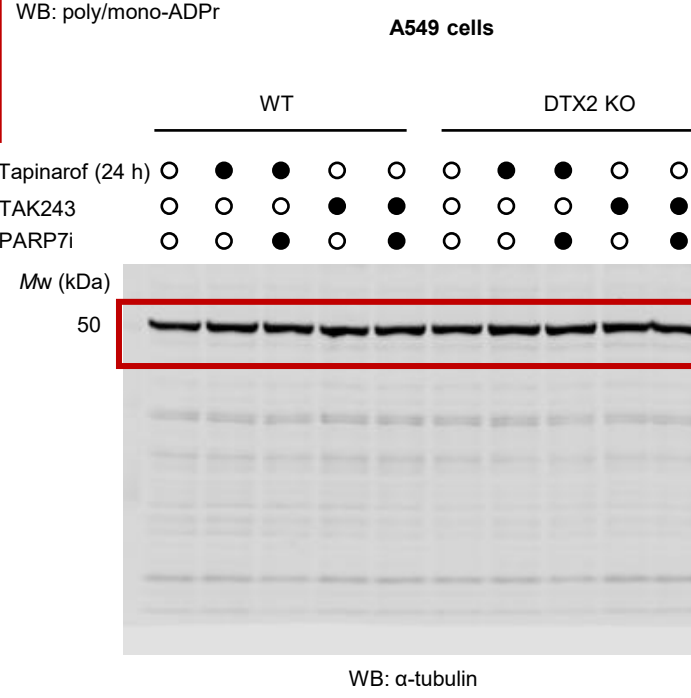

|                  | WT |   |   |   | DTX2 KO |   |   |   |   |
|------------------|----|---|---|---|---------|---|---|---|---|
| Tapinarof (24 h) | ○  | ● | ● | ○ | ○       | ○ | ● | ● | ○ |
| TAK243           | ○  | ○ | ○ | ● | ●       | ○ | ○ | ○ | ● |
| PARP7i           | ○  | ○ | ● | ○ | ●       | ○ | ○ | ● | ○ |

Supplement: Supplementary file 7 — Source data Fig. 5 [file 44318_2025_656_MOESM7_ESM.zip › Figure 5/5A/Fig. 5A.pdf]

Fig.5B

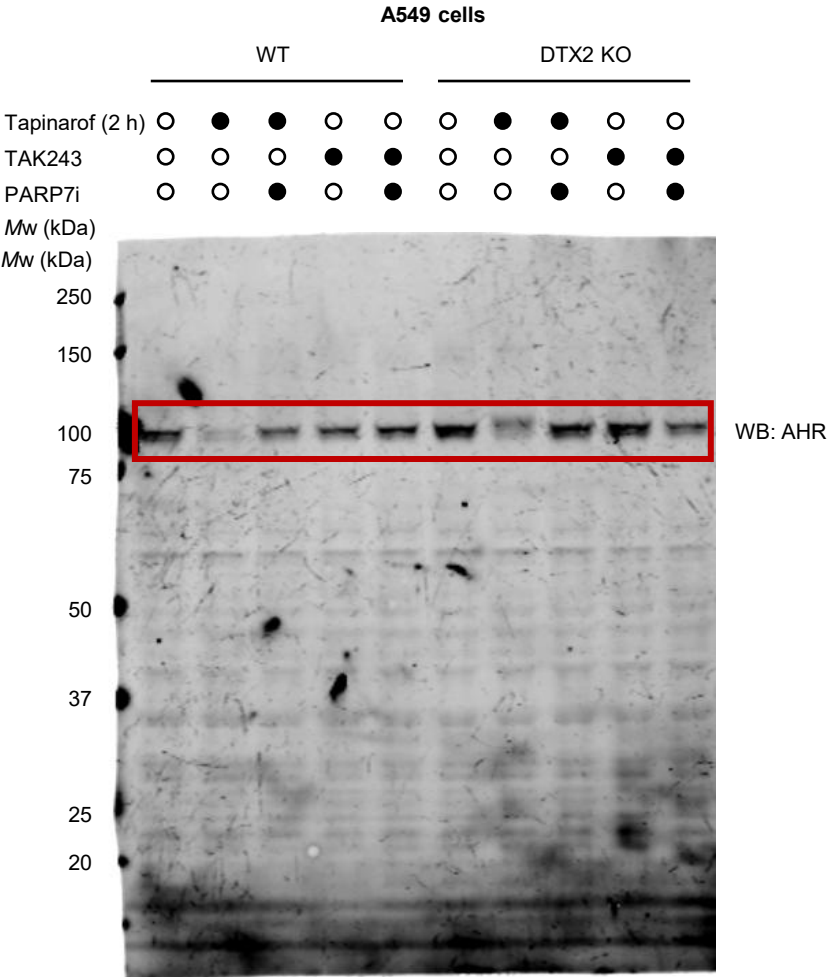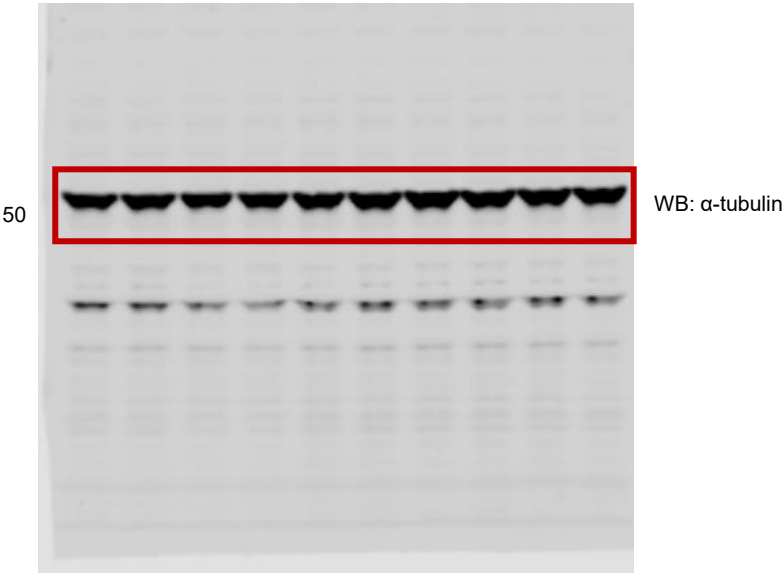

Supplement: Supplementary file 7 — Source data Fig. 5 [file 44318_2025_656_MOESM7_ESM.zip › Figure 5/5B/Fig. 5B.pdf]
