# Supplementary material for: Ubiquitin pathway blockade reveals endogenous ADP-ribosylation marking PARP7 and AHR for degradation
Source: EMBO J. 2025 Dec 1;45(1):261–77. doi: 10.1038/s44318-025-00656-1 (PMC12759070; doi:10.1038/s44318-025-00656-1)
Supplement: Supplementary file 5 — Source data Fig. 3 [file 44318_2025_656_MOESM5_ESM.zip › Figure 3/3B/Fig. 3B.pdf]

Fig. 3B

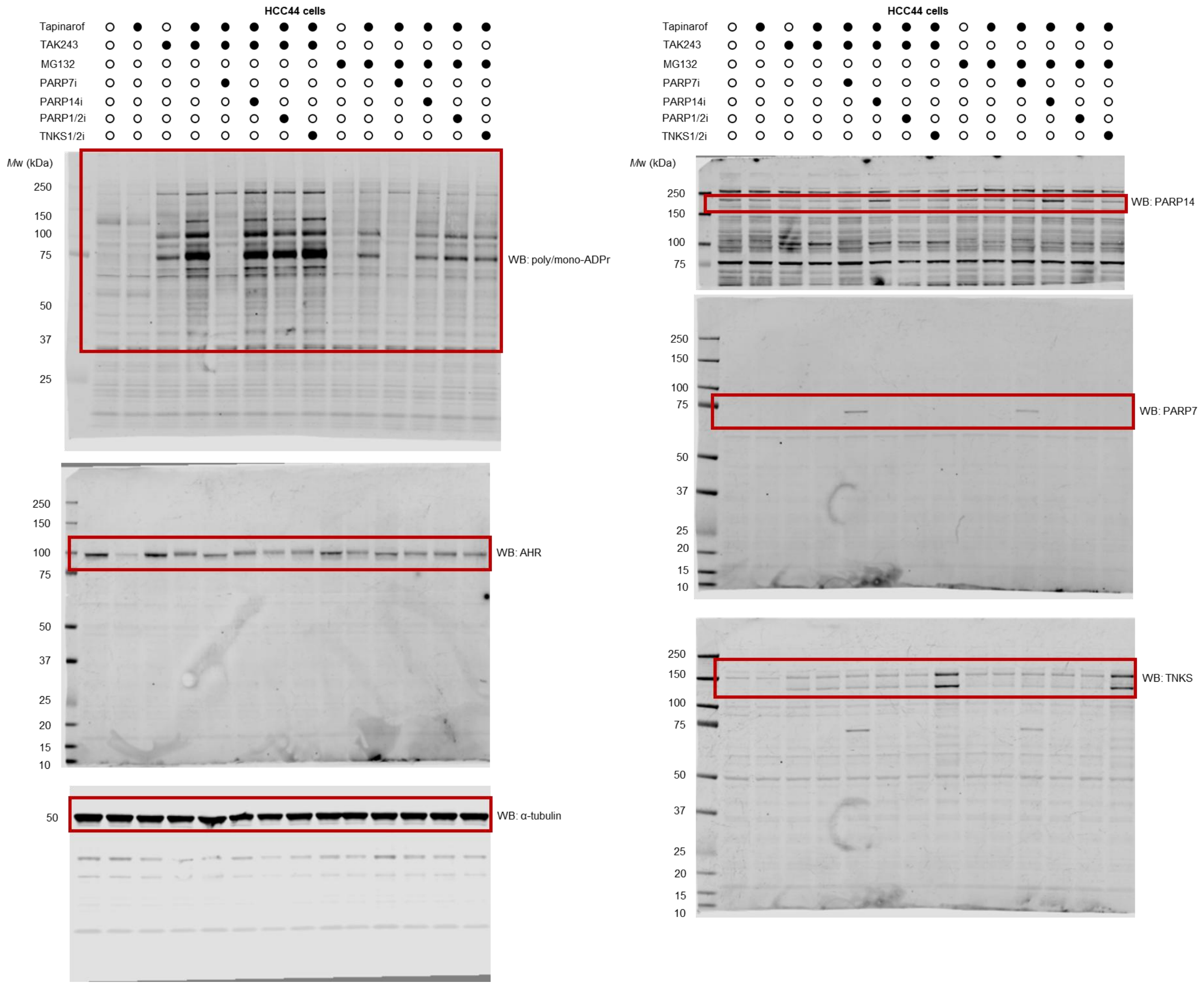

HCC44 cells
Tapinarof
TAK243
MG132
PARP7i
PARP14i
PARP1/2i
TNKS1/2i
Mw (kDa)
250
150
100
75
50
37
25
20
15
10
WB: poly/mono-ADPr
WB: AHR
WB: α-tubulin
WB: PARP14
WB: PARP7
WB: TNKS
